# Einstellungen dermatologischer Patienten gegenüber digitalen Gesundheitsinterventionen: Eine Querschnittsbefragung und Clusteranalyse

**DOI:** 10.1111/ddg.70010x

**Published:** 2026-09-08

**Authors:** Patrick Reinders, Matthias Augustin, Marina Otten

**Affiliations:** ^1^ Institut für Versorgungsforschung in Dermatologie und bei Pflegeberufen (IVDP) Universitätsklinikum Hamburg‐Eppendorf (UKE) Hamburg Deutschland

**Keywords:** Digitale Medizin, eHealth, Einstellungen, Patienten, attitudes, Digital Health, eHealth, Patients

## Abstract

**Hintergrund und Ziele:**

Ziel dieser Studie war es, die Akzeptanz digitaler Gesundheitsinterventionen (DHI) bei Patienten in der Dermatologie zu bewerten und Patientengruppen zu identifizieren.

**Patienten und Methoden:**

Eine quantitative Querschnittsbefragung zur Akzeptanz und (künftigen) Nutzung von DHI unter Patienten mit chronischen Hauterkrankungen wurde durchgeführt. Mittels explorativer Faktorenanalyse (EFA) wurden Faktoren identifiziert und die Patienten anhand eines zweistufigen Clustering‐Ansatzes in Gruppen eingeteilt.

**Ergebnisse:**

Die EFA verwendete Daten von 344 Patienten (M 52,5 Jahre, SD 14,8; 74,0 % weiblich) und ergab vier Faktoren: „Akzeptanz/Vertrauen“, „Digitale Gesundheitskompetenzen“, „Unsicherheiten“ und „Positive Auswirkungen“. Die Clusteranalyse ergab vier Gruppen: „Digitale Skeptiker“ (n = 49, 15,6 %), „Vorsichtige Anwender“ (n = 106, 33,7 %), „Digitale Enthusiasten“ (n = 98, 31,1 %) und „Anwender, unsicher über die Auswirkungen“ (n = 62, 18,0 %). Die „Digitalen Enthusiasten“ waren deutlich jünger. Die Nutzung und Akzeptanz variierten zwischen den Clustern, wobei die „Enthusiasten“ und die „Anwender, unsicher über die Auswirkungen“ die höchsten Werte aufwiesen. Insgesamt nutzten nur wenige Patienten DHI, darunter Store‐and‐Forward‐Teledermatologie (2,0–7,0 %) oder elektronische Patientenakten (6,4–19,4 %).

**Schlussfolgerungen:**

Patientengruppen mit unterschiedlichen Einstellungen gegenüber DHI wurden identifiziert. Trotz einer allgemeinen Bereitschaft zur Einführung von DHI bleibt die tatsächliche Nutzung gering. Systemische Barrieren, wie Verfügbarkeit und digitale Kompetenzen, müssen angegangen werden.

## EINLEITUNG

Die Dermatologie ist durch das weit verbreitete Auftreten von Hauterkrankungen gekennzeichnet, darunter häufige chronische Hautkrankheiten wie Akne, Psoriasis, atopische Dermatitis und Hautkrebs.^1^, ^2^ In Deutschland sind jährlich etwa 27 % der Erwachsenen von mindestens einer dermatologischen Erkrankung betroffen. Dies führt zu einer vergleichsweise hohen Fallzahl und damit zu langen Wartezeiten auf einen Termin bei einem niedergelassenen Facharzt.^3^, ^4^ Darüber hinaus wird davon ausgegangen, dass sich die Kapazitätsengpässe durch den demografischen Wandel noch weiter verschärfen werden.^4^, ^5^, ^6^, ^7^

Als Ansatz für eine nachhaltige Gesundheitsversorgung zielen digitale Gesundheitsinterventionen (digital health interventions; DHI) darauf ab, die Versorgungsqualität zu verbessern, die Effizienz zu steigern und Patienten zu stärken.^8^, ^9^ In dieser Studie beziehen sich DHI auf Gesundheitsdienste, die Informations‐ und Kommunikationstechnologien für Patienten, Verbraucher oder Angehörige der Gesundheitsberufe nutzen.^9^, ^10^ In der Dermatologie sind bereits heute vielversprechende DHI im Einsatz,^11^ darunter Anwendungen künstlicher Intelligenz bei der Diagnosestellung,^12^ Teledermatologie für Überweisungen, Diagnosen oder die Überwachung von Hautkrankheiten^13^, ^14^, ^15^ und patientenorientierte Anwendungen zur Verbesserung der Medikamenteneinnahme.^16^ Die s2k‐Leitlinie Teledermatologie aus dem Jahr 2020 unterstützt den Einsatz der Teledermatologie für bestimmte Indikationen, betont jedoch auch die Notwendigkeit von weiterer Evidenz zur Bewertung der damit verbundenen Vorteile und Risiken.^17^

Trotz der vorhandenen digitalen Gesundheitsinfrastrukturen hinkt die Digitalisierung im Gesundheitswesen in Deutschland hinter anderen westlichen Ländern hinterher.^18^ In der Dermatologie haben selbst auf dem Höhepunkt der COVID‐19‐Pandemie nur wenige Dermatologen digitale Gesundheitsinfrastrukturen in ihre Behandlung integriert.^19^, ^20^ Darüber hinaus ist die Nutzung durch Patienten mit Hauterkrankungen relativ gering, beispielsweise nutzten nur 10 % eine elektronische Patientenakte (ePA).^21^ Die Komplexität des deutschen Gesundheitssystems trägt zu geringen DHI‐Implementierungsraten bei, wobei Faktoren wie fehlende Interoperabilität, hohe Datenschutzstandards oder fehlende Anreize für Kliniken und ambulante Praxen eine Rolle spielen.^22^

Weitere Gründe für die geringe Nutzungsrate von DHI, die von Dermatologen beschrieben werden, können die geringe Akzeptanz von DHI bei Patienten und deren begrenzten digitalen Kompetenzen sein.^23^ Einige Veröffentlichungen zeigen jedoch eine ausreichende bis hohe Akzeptanz von DHI und ausreichende digitale Kompetenzen.^21^, ^24^, ^25^ Die Studien sind jedoch entweder durch eine geringe Stichprobengröße, die Konzentration auf eine dermatologische Indikation, eine strenge Auswahl der Patienten oder durch eine begrenzte Anzahl von Items zur näheren Untersuchung der Patientenakzeptanz eingeschränkt.

Mit unserer Studie möchten wir die vorhandene Literatur ergänzen, indem wir die Akzeptanz und die aktuelle/zukünftige Nutzung von DHI bei Patienten mit chronischen Hauterkrankungen untersuchen. Weitere Ziele sind die Identifizierung unterschiedlicher Patientengruppen auf der Grundlage ihrer Akzeptanz und die Untersuchung ihrer Zusammenhänge mit soziodemografischen Variablen und der Akzeptanz von DHI.

## PATIENTEN UND METHODIK

Zur Beschreibung der Studie wurde die konsensbasierte Checkliste „Checklist for Reporting of Survey Studies“ (CROSS) herangezogen.^26^ Die Checkliste ist im Online‐Supplement S1 verfügbar.

### Studiendesign und Fragebogen

Im März 2022 wurde eine anonyme Querschnittsbefragung unter Patienten mit chronischen Hauterkrankungen in Deutschland durchgeführt. Die Befragung basierte auf den Ergebnissen zuvor durchgeführter Fokusgruppen mit Dermatologen, Patienten und Pflegekräften sowie auf Erkenntnissen aus der vorhandenen Literatur. Das Ziel der Fokusgruppenstudie war es, die Akzeptanz, Barrieren und Förderfaktoren von DHI in der Dermatologie zu untersuchen.^23^ Auf der Grundlage dieser Ergebnisse entwickelten wir Kategorien und passten sie an Fragebögen aus der Literatur an.^21^, ^27^, ^28^ Dabei entwickelten wir 31 Items zur Akzeptanz (zum Beispiel „Ich könnte mir vorstellen vermehrt digitale Anwendungen für meine Erkrankung zu nutzen“). Basierend auf der systematischen Literaturrecherche^11^ wurden elf Items zur potenziellen zukünftigen Nutzung spezifischer DHI aufgenommen (zum Beispiel Teledermatologie oder elektronisches Patiententagebuch). Die Befragten bewerteten alle Items auf einer fünfstufigen Likert‐Skala (1 – stimme gar nicht zu; 5 – stimme völlig zu); die Zahlen waren während der Beantwortung nicht sichtbar. Darüber hinaus wurden in der Befragung Fragen zur aktuellen Nutzung derselben elf spezifischen DHI gestellt, die mit „Ja“, „Nein“ oder „Ich weiß nicht“ beantwortet werden konnten.

Zu den soziodemografischen Angaben gehörten Alter, Geschlecht, Bildungsstand und Postleitzahl. Letztere wurde verwendet, um den Teilnehmern zusätzliche regionale Informationen (zum Beispiel Einstufung als städtisch oder ländlich) zuzuordnen. Die Befragung umfasste auch Angaben zu den dermatologischen Erkrankungen der Patienten (wie Psoriasis, atopische Dermatitis, Vitiligo, Hautkrebs und Hidradenitis suppurativa), zur Dauer seit dem Auftreten der Symptome und zur Diagnose, zur selbst wahrgenommenen Gesundheit anhand des ersten Items des SF‐36,^29^ sowie Details zum behandelnden Arzt.

Der Fragebogen wurde mit Mitgliedern der Patientenvereinigung ‘Deutscher Psoriasis Bund e.V.’ getestet und angepasst. Die endgültigen Fragen sind im Online‐Supplement S2 zu finden.

### Studienpopulation, Rekrutierung und Dateneingabe

Um eine explorative Faktorenanalyse (EFA) durchzuführen, wie im Abschnitt zur statistischen Analyse beschrieben, strebten wir eine Mindeststichprobengröße von 150 Patienten an.^30^ Um die Verallgemeinerbarkeit unserer Ergebnisse zu verbessern, schlossen wir Patienten ein, bei denen verschiedene chronische Hauterkrankungen diagnostiziert worden waren, darunter Psoriasis, Vitiligo, Hidradenitis suppurativa, atopische Dermatitis und Hautkrebs. Die Patientenrekrutierung erfolgte online über verschiedene Selbsthilfegruppen und Patientenvertretungen, nämlich den

- *Deutscher Psoriasis Bund* für Psoriasis (circa 1700 Mitglieder per E‐Mail eingeladen),
- *Deutscher Vitiligo Bund* für Vitiligo (circa 250 Mitglieder per E‐Mail eingeladen),
- *MulleWupp e. V*. für Hidradenitis suppurativa (Beitrag in Facebook‐Gruppe mit circa 900 Mitgliedern),
- *Deutscher Allergie und Asthmabund* für atopische Dermatitis (circa 1000 Mitglieder per E‐Mail eingeladen),
- *SHG Hautkrebs Hamburg.info* für Hautkrebs (circa 400 Mitglieder per E‐Mail eingeladen),
- und *Melanom‐Info Deutschland* für Hautkrebs (Beitrag in einer Facebook‐Gruppe mit etwa 8000 Mitgliedern).

Die Online‐Befragung wurde über Unipark durchgeführt und endete 12 Wochen nachdem die erste Organisation ihre Teilnehmer eingeladen hatte. Um Mehrfachteilnahmen eines einzelnen Patienten zu verhindern, wurde die Teilnahme mithilfe des Cookie‐Systems von Unipark überwacht (keine gespeicherten IP‐Adressen).

### Ethische Genehmigung

Die Studie wurde der Lokale Psychologische Ethikkommission am Zentrum für Psychosoziale Medizin vorgelegt und genehmigt (LPEK‐0407). Sie wurde unter Einhaltung der Grundsätze der guten wissenschaftlichen Praxis und der Deklaration von Helsinki durchgeführt.^31^, ^32^

### Statistische Analyse

Nach einer deskriptiven Analyse aller soziodemografischen und geografischen Parameter wurde eine explorative Faktorenanalyse (EFA) (Rotationsmethode: Oblimin) für alle 31 Items zur Akzeptanz durchgeführt, und zwar aus zwei Hauptgründen: *(1)* Datenreduktion durch Identifizierung zugrunde liegender Faktoren und *(2)* Verwendung der Faktoren zur besseren Beschreibung der Akzeptanz bei Patienten.^33^ Die Angemessenheit für die EFA wurde mit einem Kaiser‐Meyer‐Olkin‐Kriterium (KMO) ≥ 0,8 und einem Bartlett‐Test auf Sphärizität < 0,05 angenommen. Faktoren mit einem Eigenvalue ≥ 1,0 wurden berücksichtigt. Items wurden auf der Grundlage einer substanziellen Faktorladung (<|0,4|) auf einen Faktor beibehalten, ohne starke Kreuzladung auf andere Faktoren. Eine Kreuzladung wurde als Ladung (>|0,3|) auf einen anderen Faktor definiert, wobei die Differenz zwischen der Ladung auf den Hauptfaktor und der stärksten Ladung auf einen anderen Faktor weniger als 0,2 betrug.^34^ Die interne Konsistenz der Faktoren wurde mit Cronbachs Alpha bewertet, wobei Werte > 0,7 als akzeptabel, > 0,8 als gut und > 0,9 als ausgezeichnet angesehen wurden.

Um unterschiedliche Cluster unter Patienten mit chronischen Hauterkrankungen zu erkennen, die sich in ihrer Akzeptanz unterscheiden, wurde ein zweistufiger Clustering‐Ansatz kombiniert aus hierarchischer und K‐Means‐Clusteranalyse verwendet. Die Durchschnittswerte (unter Verwendung der Likert‐Skala: 1–5) der Faktoren dienten als Eingabevariablen für beide Ansätze. Die hierarchische Clustermethode bestimmte die optimale Anzahl von Clustern und anschließend wurde auf der Grundlage dieser optimalen Anzahl eine K‐Means‐Clusteranalyse durchgeführt. Um die Unterschiede zwischen den identifizierten Clustern hinsichtlich der Durchschnittswerte zu untersuchen, wurde eine einseitige Varianzanalyse (ANOVA) durchgeführt. Um die identifizierten Cluster zu profilieren, wurde jeder Punkt aus der EFA binär codiert (1: stimme voll zu/stimme zu; 0: stimme teilweise zu, stimme nicht zu, stimme überhaupt nicht zu). Anschließend wurde eine deskriptive Analyse durchgeführt, bei der jedes Item für jeden Cluster untersucht wurde. Für kategoriale Variablen wurden Chi‐Quadrat‐Tests angewendet, um Zusammenhänge zwischen Clustern und soziodemografischen Parametern aufzudecken. Wenn mehr als 20 % der Zellen eine Anzahl von weniger als fünf aufwiesen, wurde der Exakte Fisher‐Test (FET) verwendet. Für kontinuierliche Variablen wurde die einseitige ANOVA verwendet. Ein Signifikanzniveau von 0,05 wurde verwendet und die *Missings* wurden für alle Variablen angegeben. Imputations‐ oder Gewichtungsmethoden wurden nicht angewendet. Für alle statistischen Analysen wurde SPSS Version 29 verwendet.

## ERGEBNISSE

Insgesamt nahmen 344 Patienten mit mindestens einer der vordefinierten Hauterkrankungen an der Befragung teil. Sie waren im Durchschnitt 52,5 Jahre alt (Standardabweichung [SD] 14,8), 74,0 % waren weiblich, 75,9 % lebten in einem städtischen Landkreis oder einer Stadt und 58,9 % hatten ein hohes Bildungsniveau (Tabelle 1). Die fünf untersuchten Hauterkrankungen waren Psoriasis (39,0 %), atopische Dermatitis (23,3 %), Hidradenitis suppurativa (21,2 %), Vitiligo (9,0 %) und Hautkrebs (7,6 %). Ein erheblicher Teil der Teilnehmer (65,0 %) lebte seit über 10 Jahren mit ihrer Hauterkrankung, wobei die durchschnittliche Dauer 23,9 Jahre (SD 19,5) betrug. Nur 18,0 % bewerteten ihren Gesundheitszustand als ausgezeichnet oder sehr gut, während 36,6 % ihn als mittelmäßig oder schlecht bewerteten. Die Rate der fehlenden Daten lag für alle Items unter 5,0 %, mit Ausnahme der regionalen Unterschiede (21 fehlende Daten, 6,1 %), der Bildung (18 fehlende Daten, 5,2 %) und der Zeit seit der Erstdiagnose (18 fehlende Daten, 5,2 %).

**TABELLE 1:** Soziodemografische Merkmale der gesamten Studienpopulation und der identifizierten Cluster.

	Gesamtstudienpopulation* (n = 344)	Digitale Skeptiker (Cluster 1) (n = 49)	Vorsichtige Anwender (Cluster 2) (n = 106)	Digitale Enthusiasten (Cluster 3) (n = 98)	Anwender, die sich über die Auswirkungen unsicher sind (Cluster 4) (n = 62)	p‐Wert
*Alter in Jahren, n (%)*						< 0,001
18–44	105 (31,5)	9 (18,4)	38 (35,8)	42 (44,7)	14 (23,3)	
45–64	147 (44,1)	18 (36,7)	49 (46,2)	43 (45,7)	29 (48,3)	
65+	81 (24,3)	22 (44,9)	19 (17,9)	9 (9,6)	17 (28,3)	
Fehlt	11	0	0	4	2	
Mittelwert (SD)	52,5 (14,8)	59,2 (13,0)^b^, ^c^	49,8 (14,5)^a^	47,1 (13,8)^a^, ^d^	55,5 (13,6)^c^	< 0,001
*Geschlecht, n (%)*						0,45
Weiblich	247 (74,0)	35 (72,9)	84 (80,0)	68 (71,6)	43 (70,5)	
Männlich	87 (26,0)	13 (27,1)	21 (20,0)	27 (28,4)	18 (29,5)	
Fehlend	10	1	1	3	2	
*Regionale Unterschiede, n (%)*						
Stadtgebiet oder Großstadt	245 (75,9)	38 (82,6)	77 (74,8)	66 (72,5)	46 (75,7)	0,62
Ländlicher Landkreis	78 (24,1)	8 (17,4)	26 (25,2)	25 (27,5)	14 (23,3)	
Fehlend	21	3	3	7	2	
*Bildung, n (%)*						< 0,001
Niedrig	28 (8,1)	6 (12,5)	11 (10,9)	8 (8,2)	1 (1,8)	
Mittel	106 (30,8)	20 (41,7)	43 (42,6)	21 (21,4)	15 (26,3)	
Hoch	192 (58,9)	22 (45,8)	47 (46,5)	69 (70,4)	41 (71,9)	
Fehlend	18	1	5	0	5	
*Indikation, n (%)*						
Psoriasis	134 (39,0)	23 (46,9)	37 (34,9)	26 (26,5)	31 (50,0)	0,010
Atopische Dermatitis	80 (23,3)	16 (32,7)	23 (21,7)	22 (22,4)	13 (21,0)	0,43
Vitiligo	31 (9,0)	4 (8,2)	10 (9,4)	9 (9,2)	5 (8,1)	0,98
Hautkrebs	26 (7,6)	2 (4,1)	8 (7,5)	12 (12,2)	3 (4,8)	0,23
Hidradenitis suppurativa	73 (21,2)	4 (8,2)	27 (25,5)	29 (29,6)	12 (19,4)	0,025
*Zeit seit der Erstdiagnose in Jahren, n (%)*						0,01
0	66 (21,8)	4 (8,9)	24 (24,2)	30 (30,9)	8 (12,9)	
5–9	40 (13,2)	3 (6,7)	15 (15,2)	14 (14,4)	8 (12,9)	
10	197 (65,0)	38 (84,4)	60 (60,6)	53 (54,6)	46 (74,2)	
Fehlend	18	4	7	1	0	
Mittelwert, SD	23,9 (19,5)	34,7 (21,2)^b^, ^c^	23,0 (19,7)^a^	17,8 (17,1)^a^, ^d^	26,9 (17,6)^c^	< 0,001
*Allgemeine Gesundheitswahrnehmung (SF‐36; 1 Item), n (%)*						0,20
Ausgezeichnet oder sehr gut	62 (18,0)	5 (10,2)	16 (15,1)	24 (24,5)	14 (22,6)	
Gut	152 (44,2)	23 (46,9)	48 (45,3)	35 (35,7)	30 (48,8)	
Befriedigend oder schlecht	126 (36,6)	21 (42,9)	42 (39,6)	39 (39,8)	18 (29,0)	
Fehlend	4	0	0	0	0	

*Abk*.: DHI, digitale Gesundheitsintervention; SD, Standardabweichung; SF‐36, Short Form Health Survey‐36

^a^ Unterschiedlich zu Cluster 1 (Digitale Skeptiker), p < 0,05

^b^ Unterschiedlich zu Cluster 2 (Vorsichtige Anwender), p < 0,05

^c^ Unterschiedlich zu Cluster 3 (Digitale Enthusiasten), p < 0,05

^d^ Unterschiedlich zu Cluster 4 (Anwender, die sich über die Auswirkungen unsicher sind), p < 0,05

* 29 Teilnehmer konnten aufgrund fehlender Werte der Durchschnittswerte nicht in die Clusteranalyse einbezogen werden.

### Faktoren der Akzeptanz

Die Eignung der Daten für die Durchführung einer EFA wurde durch das KMO‐Kriterium (0,90) und den Bartlett‐Test auf Sphärizität (X^2^ (df: 171) = 3335, p < 0,001) bestätigt. Es wurden vier Faktoren mit Eigenvalues größer als 1 identifiziert (Online‐Supplement S3), wobei die berechneten Cronbach‐Alpha‐Werte (0,89, 0,82, 0,79 und 0,91) auf eine zumindest akzeptable interne Konsistenz hindeuten. Die folgenden Faktornamen wurden vergeben:

- Faktor 1: „Akzeptanz von DHI und Vertrauen“ umfasste 10 Items,
- Faktor 2: „Digitale Gesundheitskompetenzen der Patienten“ umfasste 6 Items,
- Faktor 3: „Unsicherheiten bezogen auf DHI“ umfasste 5 Items,
- Faktor 4: „Positive Auswirkungen von DHI“ umfasste 5 Items.

Die Durchschnittswerte konnten zwischen 1 und 5 liegen, wobei höhere Werte eine stärkere Übereinstimmung mit den zugrunde liegenden Items anzeigten. In den Faktoren 1, 2 und 4 spiegeln höhere Werte eine positivere Wahrnehmung wider, während in Faktor 3 höhere Werte eine negativere Wahrnehmung widerspiegeln, entsprechend den Konnotationen der zugrunde liegenden Items. Die Durchschnittswerte für Faktor 1 und Faktor 4 waren moderat bis hoch (Faktor 1: Mittelwert [M] 3,9, SD 0,7; Faktor 4: M 3,6, SD 1,0), während der Mittelwert für Faktor 2 hoch war (M 4,0, SD 0,7) und der für Faktor 3 niedrig (M 2,4, SD 0,4) (Tabelle 2).

**TABELLE 2:** Unterschiede in den identifizierten Subskalen zwischen den Clustern.

	Gesamtstudienpopulation (n = 344)^*^	Digitale Skeptiker (Cluster 1) (n = 49)	Vorsichtige Anwender (Cluster 2) (n = 106)	Digitale Enthusiasten (Cluster 3) (n = 98)	Anwender, die sich über die Auswirkungen unsicher sind (Cluster 4) (n = 62)	p‐Wert (ANOVA)
Mittelwert, Standardabweichung						
Faktor 1: Akzeptanz von DHI und Vertrauen	3,9 (0,7)	2,8 (0,6)^b^, ^c^, ^d^	3,9 (0,4)^a^, ^c^	4,5 (0,3)^a^, ^b^, ^d^	3,9 (0,6)^a^, ^c^	< 0,001
Faktor 2: Digitale Gesundheitskompetenzen der Patienten	4,0 (0,7)	3,2 (0,7)^b^, ^c^, ^d^	3,8 (0,5)^a^, ^c^, ^d^	4,5 (0,4)^a^, ^b^	4,3 (0,5)^a^, ^b^	< 0,001
Faktor 3: Unsicherheiten bezogen auf DHI	2,4 (0,4)	3,0 (0,6)^c^, ^d^	2,9 (0,5)^c^, ^d^	1,8 (0,5)^a^, ^b^	1,7 (0,4)^a^, ^b^	< 0,001
Faktor 4: Positive Auswirkungen von DHI	3,6 (1,0)	2,2 (0,6)^b^, ^c^, ^d^	3,8 (0,5)^a^, ^c^, ^d^	4,5 (0,5)^a^, ^b^, ^d^	2,8 (0,6)^a^, ^b^, ^c^	< 0,001

*Abk*.: ANOVA, Varianzanalyse; DHI, digitale Gesundheitsintervention

^a^ Unterschiedlich zu Cluster 1 (Digitale Skeptiker), p < 0,05

^b^ Unterschiedlich zu Cluster 2 (vorsichtige Anwender), p < 0,05

^c^ Unterschiedlich zu Cluster 3 (Digitalbegeisterte), p < 0,05

^d^ Unterschiedlich zu Cluster 4 (Anwender, die sich über die Auswirkungen unsicher sind), p < 0,05

* 29 Teilnehmer konnten aufgrund fehlender Werte der Mittelwerte nicht in die Clusteranalyse einbezogen werden.

### Patientencluster basierend auf deren Akzeptanz

Die optimale Lösung, die durch hierarchische Clusteranalyse ermittelt wurde, ergab eine Klassifizierung in vier Cluster. Nachfolgende k‐Means‐Analysen (ANOVA und Post‐hoc‐Tests) bestätigten signifikante Unterschiede über alle vier Subskalen hinweg (Tabelle 2).

Cluster 1 (n = 49; 15,6 %) zeigte moderate Ratings für Akzeptanz und Vertrauen (Faktor 1; M 2,8, SD 0,6) und Kompetenz (Faktor 2: M 3,2, SD 0,7) sowie geringe Erwartungen hinsichtlich einer positiven Wirkung von DHI (Faktor 4: M 2,2, SD 0,6). Alle Faktoren wurden im Vergleich zu den anderen drei Clustern signifikant niedriger bewertet. Darüber hinaus waren die Unsicherheiten mit DHI moderat (Faktor 3: M 3,0, SD 0,6) und signifikant höher als in den Clustern 3 und 4.

Cluster 2 (n = 106; 33,7 %) wies moderate bis hohe Durchschnittswerte in Bezug auf Akzeptanz und Vertrauen (Faktor 1: M 3,9, SD 0,5), Kompetenzen (Faktor 2: M 3,8, SD 0,5) und Erwartungen hinsichtlich positiver Auswirkungen (Faktor 4: M 3,8, SD 0,5) auf. Die Akzeptanz und das Vertrauen waren deutlich geringer als in Cluster 3 und die Kompetenzen und positiven Erwartungen waren im Vergleich zu den Clustern 3 und 4 geringer. Die Unsicherheiten bezogen auf DHI (Faktor 3: M 2,9, SD 0,5) waren moderat und deutlich höher als in den Clustern 3 und 4.

Cluster 3 (n = 98; 31,1 %) wies unter allen Clustern die höchsten Ratings hinsichtlich Akzeptanz und Vertrauen (Faktor 1: M 4,5, SD 0,3) sowie hinsichtlich der Erwartungen an eine positive Wirkung von DHI (Faktor 4: M 4,5, SD 0,5) auf. Auch die digitalen Kompetenzen wurden hoch bewertet (Faktor 2: M 4,5; SD 0,4), während die Unsicherheiten gering waren (Faktor 3: M 1,8, SD 0,5). Beide Mittelwerte lagen deutlich über denen der Cluster 1 und 2.

Cluster 4 (n = 62; 18,0 %) ähnelte Cluster 2 in Bezug auf die Akzeptanz von DHI und das Vertrauen (Faktor 1: M 3,9; SD 0,6) und Cluster 3 in Bezug auf Kompetenzen und Unsicherheiten (Faktor 2: M 4,3, SD 0,5; Faktor 3: M 1,7, SD 0,5). Allerdings hatte Cluster 4 (Faktor 4: M 2,8, SD 0,6) eine deutlich geringere Wahrnehmung der potenziellen positiven Auswirkungen von DHI als die Cluster 2 (M 3,8, SD 0,5) und 3 (M 4,5, SD 0,5).

Der Übersichtlichkeit halber bezeichnen wir die Cluster 1, 2, 3 und 4 in den folgenden Abschnitten als „Digitale Skeptiker”, „Vorsichtige Anwender”, „Digitale Enthusiasten” und „Anwender, die sich über die Auswirkungen unsicher sind”.

### Soziodemografische und gesundheitsspezifische Unterschiede der Cluster

In Bezug auf die soziodemografischen Parameter ergab die Clusteranalyse signifikante Unterschiede zwischen den identifizierten Gruppen (Tabelle 1). Die „Digitalen Enthusiasten“ (M 47,1 Jahre; SD 13,8) waren deutlich jünger als die „Digitalen Skeptiker“ (M 59,2 Jahre; SD 13,0) und die „Anwender mit Unsicherheit bezogen auf die Auswirkungen von DHI“ (M 55,5 Jahre; SD 13,6). Die „Vorsichtigen Anwender“ waren jünger als die „Digitalen Skeptiker“ (M 49,8; SD 14,5 vs. 59,2 Jahre; SD 13,0). Es wurden keine statistisch signifikanten Geschlechts‐ oder geografischen Unterschiede festgestellt. Ein signifikanter Zusammenhang wurde beim Bildungsniveau gefunden, wobei „Digitale Enthusiasten“ und „Anwender, die sich über die Auswirkungen unsicher sind“ ein höheres Bildungsniveau aufwiesen (70,4 % beziehungsweise 71,9 %) als „Digitale Skeptiker“ und „Vorsichtige Anwender“ (45,8 % beziehungsweise 46,5 %).

Ein signifikanter Zusammenhang wurde zwischen der Clusterzuordnung von Psoriasis und Hidradenitis suppurativa aufgedeckt. Teilnehmer mit einer Psoriasis‐Diagnose waren häufiger in den Clustern „Digitale Skeptiker” (46,9 %) und „Anwender, die sich über die Auswirkungen unsicher sind” (50,0 %) vertreten, während diejenigen mit einer Hidradenitis‐suppurativa‐Diagnose einen größeren Anteil in den Clustern „Vorsichtige Anwender” (25,5 %) und „Digitale Enthusiasten” (29,6 %) hatten. Die Zeit seit der Erstdiagnose variierte erheblich zwischen den vier Clustern. Insbesondere die „Digitalen Skeptiker“ hatten eine signifikant längere Zeit seit der Erstdiagnose (M 34,7 Jahre; SD 21,2) im Vergleich zu den „Vorsichtigen Anwendern“ (M 23,0 Jahre; SD 19,7) und den „Digitalen Enthusiasten“ (M 17,8 Jahre; SD 17,1) (Tabelle 1). Darüber hinaus war die Zeit seit der Diagnose bei den „Anwendern, die sich über die Auswirkungen unsicher sind“ signifikant länger als bei den „Digitalen Enthusiasten“ (M 26,9 Jahre; SD 16,6 vs. 17,8; SD 17,1).

### Akzeptanz von DHI innerhalb der identifizierten Cluster

**ABBILDUNG 1:**
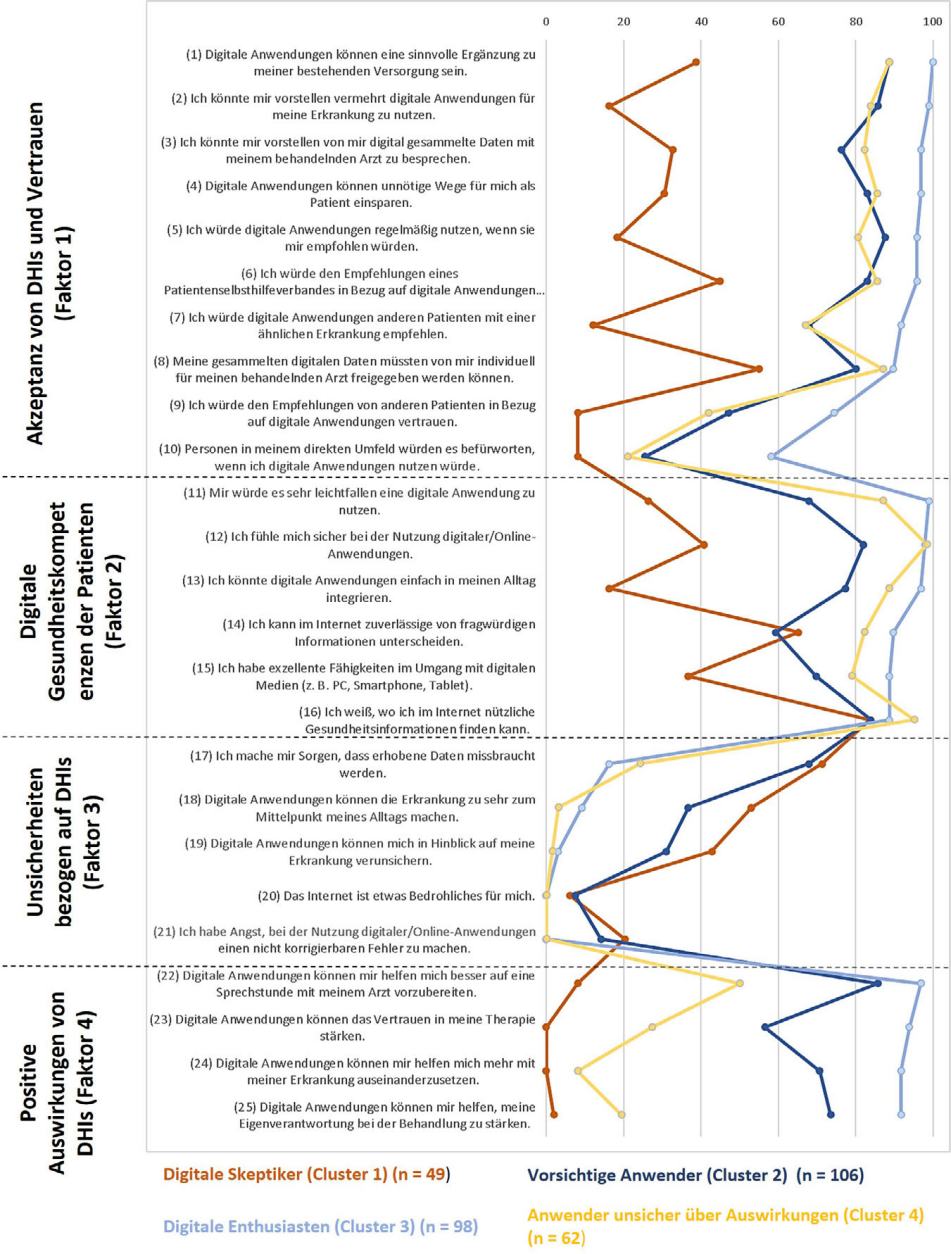
Cluster‐Profilanalyse auf Itemebene.

Die folgenden Absätze enthalten eine detaillierte Beschreibung der Items für jede der vier Cluster hinsichtlich ihrer Akzeptanz gegenüber DHI aufzeigen. Eine umfassende Zusammenfassung der Ratings für alle Items ist in Abbildung 1 dargestellt. Eine Übersicht über die gesamte Gruppe (N = 344) ist im Online‐Supplement S5 (Tabelle S7) zu finden.

„Digitale Skeptiker” zeigten niedrige Zustimmung bei den Items innerhalb von Faktor 1, die zwischen 8,2 % (Item 9: Vertrauen in Empfehlungen anderer Patienten) und 55,1 % (Item 8: Weitergabe von Daten an meinen behandelnden Arzt) lagen (Abbildung 1; Faktor 1). In Bezug auf Faktor 2 gaben die Teilnehmer selbst an, über geringe digitale Kompetenzen zu verfügen (Item 11: 26,5 %), aber auch über die Fähigkeit, nützliche Online‐Informationen zu finden (Item 16: 83,7 %) und zuverlässige von fragwürdigen Informationen zu unterscheiden (Item 14: 65,3 %) (Abbildung 1; Faktor 2). „Digitale Skeptiker” äußerten Bedenken hinsichtlich des potenziellen Missbrauchs ihrer Daten (Item 17: 71,3 %) (Abbildung 1; Faktor 3). Die Gruppe fühlte sich jedoch nicht vom Internet eingeschüchtert (Item 28: 14,2 %) (Abbildung 1; Faktor 3). Die Bewertungen der positiven Auswirkungen von DHI waren niedrig. Dabei erwartete niemand in dieser Gruppe, dass DHI das Vertrauen in die Therapie ihrer Krankheit stärken würden (Item 23: 0,0 %) (Abbildung 1; Faktor 4).

Die „Vorsichtigen Anwender” und die „Anwender, die sich über die Auswirkungen unsicher sind” wiesen hohe Bewertungen in Faktor 1 auf. Beide Gruppen waren der Meinung, dass die Anwendungen eine nützliche Ergänzung zur bestehenden Versorgung sein könnten (Item 1; „Vorsichtige Anwender”: 88,7 %; „Anwender, die sich über die Auswirkungen unsicher sind”: 88,7 %), waren sich jedoch unsicher, ob sie in ihrem unmittelbaren Umfeld Unterstützung für die Nutzung von DHI erhalten würden (Item 11: „Vorsichtige Anwender”: 25,5 %, „Anwender, die sich über die Auswirkungen unsicher sind”: 21,0 %). Bei den „Anwendern, die sich über die Auswirkungen unsicher sind” waren die Bewertungen der Kompetenz‐Items in Faktor 2 (Abbildung 1) hoch, während die „Vorsichtigen Anwender” eine geringere Zustimmung zu den Items in diesem Faktor zeigten. Beispielsweise schätzten sich 59,4 % der „Vorsichtigen Anwender” in der Lage, zuverlässige von fragwürdigen Daten zu unterscheiden (Item 14), während 82,3 % der „Anwender, die sich über die Auswirkungen unsicher sind” dieser Aussage zustimmten (Abbildung 1; Faktor 2). In Faktor 3 äußerten die „Vorsichtigen Anwender” erhebliche Bedenken hinsichtlich eines möglichen Datenmissbrauchs (67,9 %; Punkt 17) im Vergleich zu den „Anwendern, die sich über die Auswirkungen unsicher sind“ (22,3 %). Insgesamt zeigte diese letztere Gruppe niedrige Zustimmungsraten über den gesamten Faktor 3 hinweg. Beispielsweise stimmten nur 1,6 % der Aussage zu, dass DHI ihnen ein Gefühl der Unsicherheit in Bezug auf ihre Krankheit vermitteln könnten (Item 19). Im Gegensatz dazu zeigten die „Vorsichtigen Anwender” hinsichtlich der positiven Auswirkungen von DHI (Abbildung 1; Faktor 4) höhere Zustimmungsraten, die von 56,6 % (Item 23: Stärkung des Vertrauens in die Therapie) bis 85,8 % (Item 22: bessere Vorbereitung auf Arztgespräche) reichten. „Anwender, die sich über die Auswirkungen unsicher sind” gaben niedrigere Zustimmungsraten an, die zwischen 8,1 % (Item 24: Unterstützung bei der Bewältigung ihrer Krankheit) und 50,0 % (Item 22: Stärkung des Vertrauens in die Therapie) lagen.

Die „Digitalen Enthusiasten“ zeigten in allen Faktoren hohe Akzeptanzwerte (Abbildung 1). Die Zustimmungsraten für die meisten Items in den Faktoren 1, 2 und 4 lagen zwischen 85 % und 100 %. Eine Ausnahme bildete Faktor 1: 74,5 % vertrauten den Empfehlungen anderer Patienten (Item 9), und 58,2 % glaubten, dass Menschen in ihrem Umfeld digitale Gesundheitsinterventionen unterstützen würden (Item 11). Die Bewertungen der Items unter „Unsicherheiten bezogen auf DHI“ (Abbildung 1; Faktor 3) waren im Allgemeinen niedrig; so äußerte beispielsweise nur eine Minderheit Bedenken hinsichtlich eines möglichen Datenmissbrauchs (Item 17: 16,3 %).

Insgesamt stimmte die Mehrheit der Teilnehmer aller Cluster zu, dass sie ihrem behandelnden Arzt Zugriff auf ihre Daten gewähren sollten (Item 8; Faktor 1; Bereich: 55,1 %–89,9 %) und dass sie wissen, wo sie online nützliche Gesundheitsinformationen finden können (Item 16; Faktor 3; Bereich: 83,7 %–95,2 %).

### Nutzung von DHI innerhalb der identifizierten Cluster

**ABBILDUNG 2:**
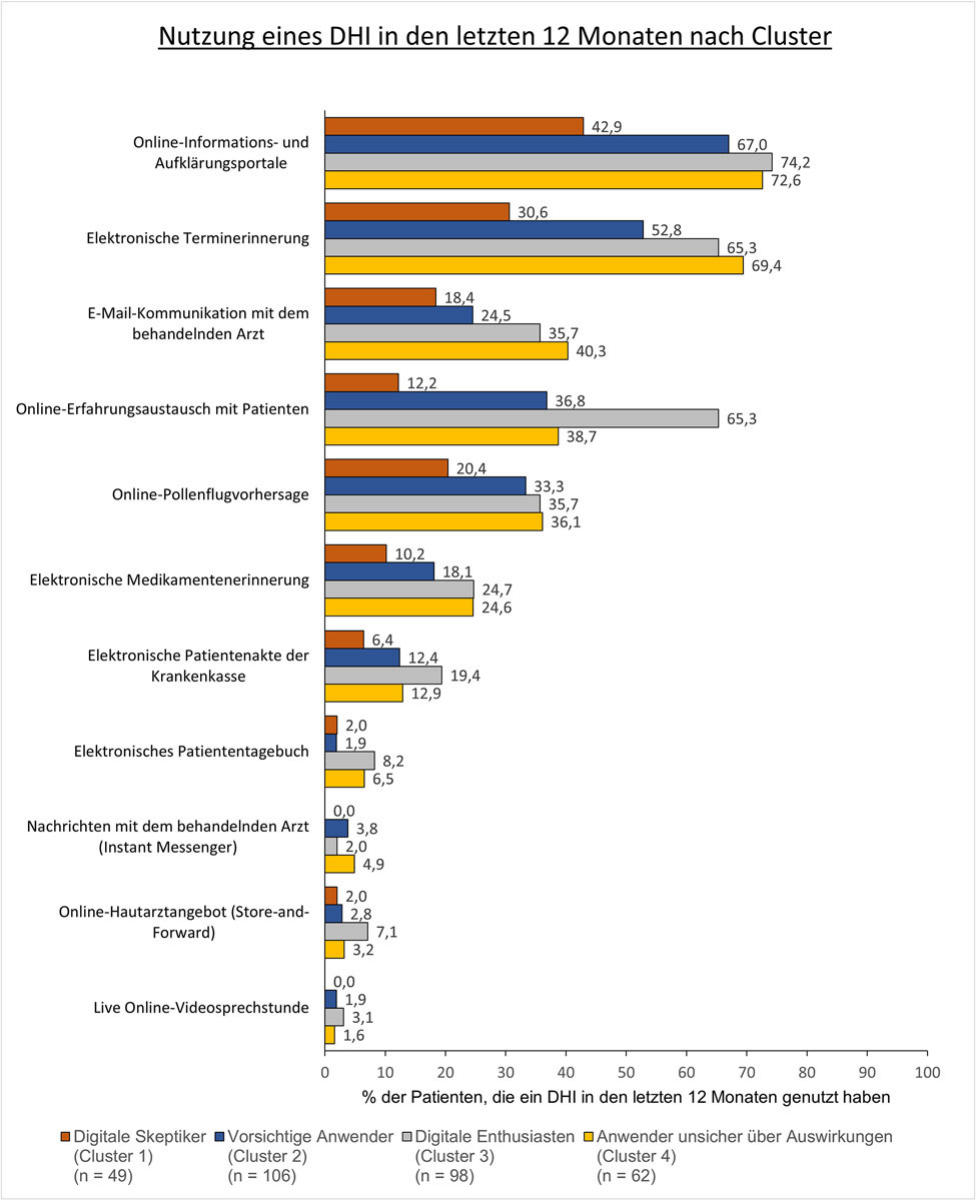
Nutzung digitaler Gesundheitsinterventionen in den letzten 12 Monaten nach Cluster.

Insgesamt war die Nutzung von DHI begrenzt, nur vier DHI – Online‐Pollenflugvorhersage (31,4 %), Online‐Austausch zwischen Patienten (40,1 %), Terminerinnerungen (53,5 %) und Informationsportale (63,7 %) – wurden von mindestens einem Drittel der Gesamtgruppe genutzt. Besonders niedrige Nutzungsraten wurden bei der Videosprechstunde (2,1 %) und der Store‐and‐Forward (S&F)‐Teledermatologie (4,1 %) beobachtet (Online‐Supplement 5; Tabelle S8). „Digitale Skeptiker” wiesen durchweg niedrige Nutzungsraten für alle DHI auf. Im Gegensatz dazu hatten sowohl „Anwender, die sich über die Auswirkungen unsicher sind” als auch „Digitale Enthusiasten” die höchsten Nutzungsraten (Abbildung 2). Beispielsweise nutzten 72,6 % („Anwender, die sich über die Auswirkungen unsicher sind”) und 74,2 % („Digitale Enthusiasten”) Online‐Informationsportale. „Vorsichtige Anwender“ wiesen bei bestimmten DHI, wie zum Beispiel Online‐Anwendungen für den Patientenaustausch (38,7 % vs. 36,8 %), vergleichbare Nutzungsraten wie „Anwender, die sich über die Auswirkungen unsicher sind“ auf, bei anderen, wie zum Beispiel der E‐Mail‐Kommunikation mit behandelnden Ärzten (40,3 % versus 24,5 %), jedoch eine deutlich geringere Nutzung. Digitale Gesundheitsinterventionen, die die Kommunikation mit Ärzten erleichtern, mit Ausnahme von E‐Mails, wurden im Allgemeinen selten genutzt. So wurde beispielsweise die S&F‐Teledermatologie von 7,1 % der „Digitalen Enthusiasten“ und 2,0 % der „Digitalen Skeptiker“ genutzt. Auch die offizielle ePA wurde in allen Clustern nur selten verwendet, von 6,4 % bei den „Digitalen Skeptikern” bis zu 19,4 % bei den „Digitalen Enthusiasten”.

### Potenzielle zukünftige Nutzung von DHI innerhalb der identifizierten Cluster

**ABBILDUNG 3:**
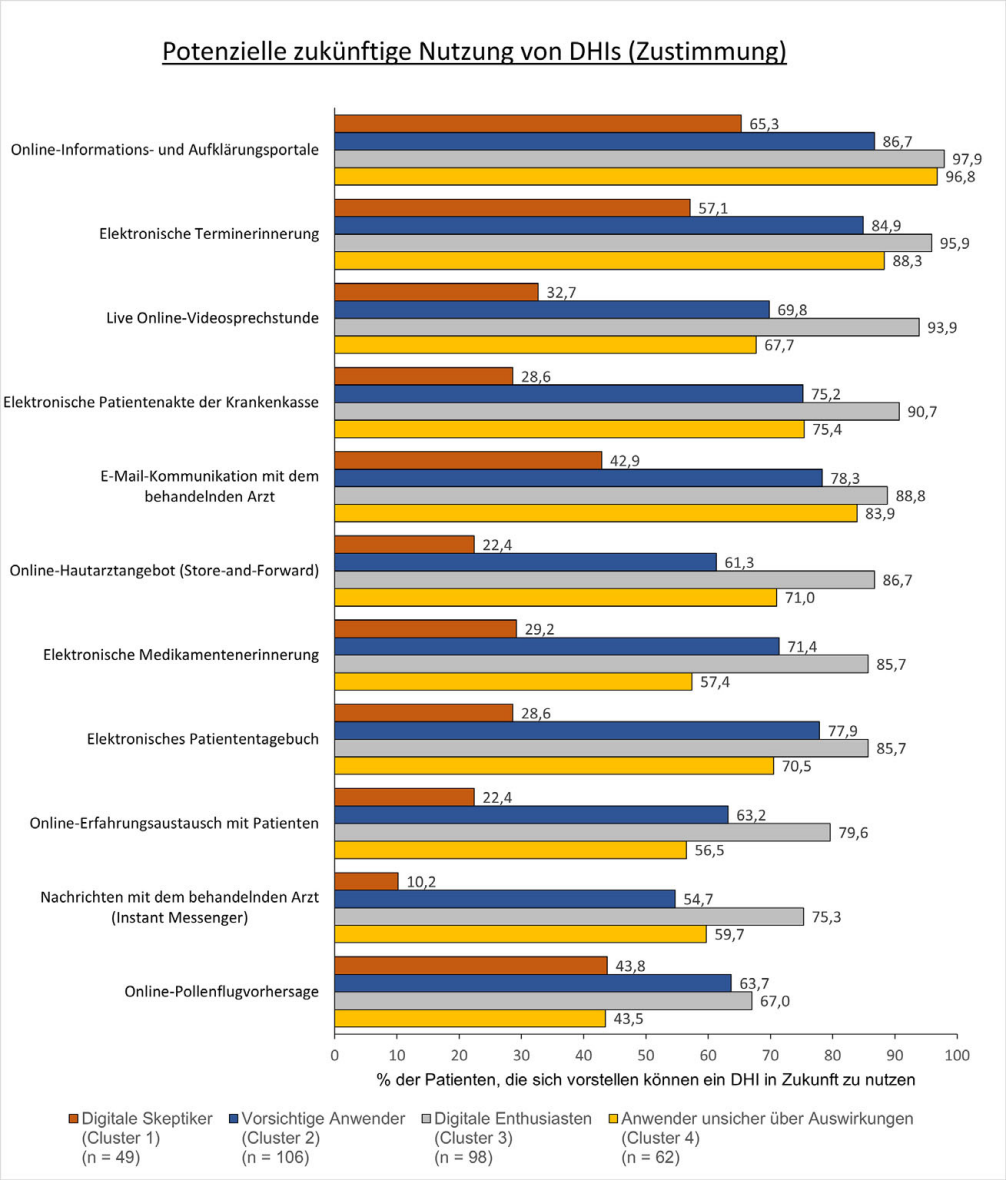
Potenzielle zukünftige Nutzung digitaler Gesundheitsinterventionen.

Für eine potenzielle zukünftige Nutzung gaben mindestens 50 % der Patienten an, dass sie die Nutzung jeder der elf DHI häufiger in Betracht ziehen würden, wobei die Spanne von 52,3 % für *Instant Messaging* mit behandelnden Ärzten bis zu 85,5 % für Online‐Informations‐ und Bildungsportale reichte (Online‐Supplement S5; Tabelle S9). Betrachtet man nur die „Digitalen Skeptiker”, so zeigte nur etwa ein Drittel Interesse daran sieben der elf DHI häufiger zu nutzen. Ausnahmen bildeten Bildungsportale (65,3 %), Terminerinnerungen (57,1 %), die Online‐Pollenflugvorhersage (43,8 %) und die Kommunikation per E‐Mail (42,9 %). Im Gegensatz dazu äußerte die klare Mehrheit innerhalb der drei Anwendercluster (Cluster 2–4) die Bereitschaft, digitale Gesundheitsdienste häufiger zu nutzen (Abbildung 3). Bei fünf der elf vorgestellten digitalen Gesundheitsdienste zeigten mindestens zwei Drittel innerhalb jedes Anwenderclusters Offenheit für eine verstärkte Nutzung. Die DHI mit den höchsten Zustimmungsraten unter den Anwendern waren Online‐Informations‐ und Bildungsportale („Vorsichtige Anwender”: 86,7 % bis „Digitale Enthusiasten”: 97,9 %), Terminerinnerungen („Vorsichtige Anwender”: 84,9 % bis „Digitale Enthusiasten”: 95,9 %) und die ePA („Vorsichtige Anwender”: 75,2 % bis „Digitale Enthusiasten” 90,7 %). Innerhalb der Gruppe der „Digitalen Enthusiasten“ lagen die Zustimmungsraten für zehn der elf vorgestellten DHI durchweg über 75 % und erreichten sogar 97,9 % für die potenzielle zukünftige Nutzung von Bildungsportalen zu ihrer Erkrankung.

## DISKUSSION

Das Ziel dieser Studie war es, den Grad der Akzeptanz sowie die aktuelle und potenzielle zukünftige Nutzung von DHI bei Patienten mit Hauterkrankungen zu untersuchen. Darüber hinaus wurden Cluster anhand ihrer Akzeptanz sowie ihrer Zusammenhänge zwischen soziodemografischen Merkmalen und der aktuellen und zukünftigen Nutzung von DHI identifiziert. Eine Mehrheit der Patienten und drei von vier identifizierten Clustern („Vorsichtige Anwender“, „Digitale Enthusiasten“, „Anwender, die sich über die Auswirkungen unsicher sind“) äußerte die Bereitschaft, DHI in Zukunft häufiger zu nutzen. Darüber hinaus verfügte die Gruppe der „Digitalen Enthusiasten“ über ausreichende digitale Kompetenzen und zeigte nur geringe Unsicherheiten im Umgang mit digitalen Gesundheitsdiensten. Die „Vorsichtigen Anwender“ zeigten ebenfalls eine positive Einstellung gegenüber DHI, äußerten jedoch Bedenken hinsichtlich des Datenmissbrauchs. Die „Anwender, die sich über die Auswirkungen unsicher sind“ erwarteten keine positiven Auswirkungen von DHI auf die Behandlung ihrer Erkrankung. Die „Digitalen Skeptiker“, zeigten einen allgemeinen Widerwillen gegen die Nutzung von DHI, verfügte über begrenzte digitale Kompetenzen und sah nur einen geringen Nutzen. Selbst unter den Patienten, die offen für die Nutzung von DHI waren, blieben die aktuellen Nutzungsraten für bestehende Interventionen wie Teledermatologie, ePA und elektronische Patiententagebücher relativ gering.

Unsere Ergebnisse unterstreichen das große Interesse von Patienten mit Hauterkrankungen an der Nutzung von DHI, aber beispielsweise haben nur 7,1 % der digital affinsten Cluster bereits S&F‐Teledermatologie genutzt. Möglicherweise gibt es systemische Barrieren wie die begrenzte Verfügbarkeit von Dienstleistungen. So bot selbst auf dem Höhepunkt der COVID‐19‐Pandemie nur eine Minderheit der Dermatologen in Deutschland (21 %) ihren Patienten S&F‐Teledermatologie an.^19^, ^20^ Darüber hinaus sind S&F‐Teledermatologie‐Lösungen derzeit nicht für chronisch kranke Patienten konzipiert, sondern konzentrieren sich auf die Erstuntersuchung von Patienten.^15^, ^35^, ^36^ Was andere DHI betrifft, so war nur eine Minderheit der in der medizinischen Literatur entdeckten Instrumente speziell für Patienten mit Hautkrankheiten konzipiert,^11^ und nur 2 % der krankheitsspezifischen Gesundheits‐Apps wurden für dermatologische Zwecke entwickelt.^37^

Interessanterweise führen viele Ärzte ihre Zurückhaltung bei der Einführung von DHI auf ein vermeintliches Desinteresse der Patienten zurück.^38^, ^39^ Unsere Daten zeigen jedoch, dass viele Patienten mit chronischen Hauterkrankungen die Nutzung dieser Dienste befürworten würden. Eine mögliche Erklärung für diese Diskrepanz könnte die unzureichende Integration der aktuellen DHI in die bestehende Versorgung sein, was dazu führt, dass Patienten und Ärzte die Standardversorgung bevorzugen. DHI, die die Kontinuität der Versorgung gewährleisten, gleichzeitig Wartezeiten verkürzen und sich nahtlos in die bestehende Versorgung integrieren lassen, werden jedoch von Patienten eher bevorzugt.^40^, ^41^

Unsicherheiten in Bezug auf DHI, insbesondere hinsichtlich des Datenmissbrauchs, stellten für die „Vorsichtigen Anwender” und „Digitalen Skeptiker” eine weitere bedeutende Barriere dar. Der negative Zusammenhang wurde bereits in früheren Untersuchungen festgestellt.^42^, ^43^, ^44^ Um die Akzeptanz von DHI zu verbessern, muss nicht nur die Datensicherheit und der Datenschutz gewährleistet werden, sondern die Patienten müssen auch aktiv über die Sicherheitsmaßnahmen und die Verwendung ihrer Daten informiert werden.

Interessant ist die Entdeckung einer Patientengruppe mit einer hohen Absicht zur Nutzung von DHI („Anwender unsicher über die Auswirkungen”), die über ausreichende digitale Kompetenzen verfügt und geringe Unsicherheiten bezogen auf DHI aufweist. Dennoch erwartet sie nur geringe Auswirkungen von DHI auf ihre Versorgung. Die Leistungserwartung ist in unserer Studie mit dem Titel „Positive Auswirkungen von DHI“ oft der zuverlässigste Prädiktor für die Akzeptanz von DHI.^27^ Diese Gruppe, in der 74 % der Patienten seit über 10 Jahren an einer Hauterkrankung leiden, hat sich möglicherweise bereits an die Anforderungen ihrer chronischen Hauterkrankungen angepasst, was zu geringeren Erwartungen an den zusätzlichen Nutzen von DHI führt.

Unsere Untersuchung hat einen *Second‐Order Digital Divide* identifiziert, der sich durch Unterschiede in den digitalen Kompetenzen, der Akzeptanz und der Nutzung auszeichnet und nicht durch Unterschiede in der Infrastruktur und den Zugangsgeräten (zum Beispiel Smartphones und Computer).^45^ In Übereinstimmung mit früheren Studien^46^, ^47^ unterstreichen unsere Ergebnisse, dass ein fortgeschrittenes Alter und ein niedrigerer Bildungsstand mit einer geringeren Nutzung von DHI verbunden waren. Diese Erkenntnis ist besorgniserregend, da der Zugang zu und die Nutzung von digitaler Gesundheit ein zunehmend wichtiger Faktor für die Gesundheit ist, insbesondere seit der COVID‐19‐Pandemie.^46^, ^48^ Einige Forscher haben sogar einen umfassenden Rahmen für digitale Gesundheitsgerechtigkeit vorgeschlagen.^49^ Mögliche Maßnahmen umfassen die Einbeziehung von Patienten unterschiedlichen Alters und Bildungsstands in die Entwicklung von DHI, die Gewährleistung der Nutzbarkeit von DHI für marginalisierte Bevölkerungsgruppen (zum Beispiel vereinfachte App‐Modi), die Schulung von Fachkräften im Gesundheitswesen und die Bereitstellung von Programmen zur Förderung der digitalen Kompetenz in lokalen Gemeinschaften, beispielsweise durch Bibliotheken.^48^, ^49^ Krankenversicherungen könnten solche Bemühungen unterstützen, da sie bereits die Verantwortung haben, digitale Gesundheitsinstrumente zu fördern und die digitale Kompetenz zu verbessern, um deren angemessene Nutzung sicherzustellen.^50^ Weitere Daten sind unerlässlich, um den Zusammenhang zwischen digitalen Ungleichheiten und gesundheitlichen Ungleichheiten besser zu verstehen und die Entwicklung wirksamer Gegenmaßnahmen zu ermöglichen.

Die durchgeführte Querschnittsbefragung weist mehrere Limitationen auf. Die Patienten wurden über Patientenorganisationen zur Teilnahme eingeladen. Forschungsergebnissen zufolge kann diese Patientengruppe eine größere Krankheitslast haben, über ein höheres Bildungsniveau verfügen, mehr Informationen erhalten und ein umfassenderes Verständnis ihrer Krankheit haben.^51^ Daher sind sie möglicherweise nicht repräsentativ für die breitere Bevölkerung, die von chronischen Hauterkrankungen betroffen ist. Die Studie konzentrierte sich auch auf Personen mit chronischen Hauterkrankungen von eher hohem Schweregrad. Die Ergebnisse berücksichtigen möglicherweise keine akuten und leichten Hauterkrankungen. Darüber hinaus nahmen an unserer Studie im Vergleich zur allgemeinen Bevölkerung mit Hauterkrankungen mehr Frauen und ältere Teilnehmer teil, was auch für jede der untersuchten Indikationen beobachtbar war (Online‐Supplement S4). Es ist bekannt, dass es zu einer Verzerrung kommen kann, da Patienten mit einer größeren Affinität zum Internet, insbesondere aufgrund der Rekrutierung per E‐Mail und über soziale Medien, möglicherweise an der Befragung teilgenommen haben. Unter Berücksichtigung aller Aspekte kann die tatsächliche Größe der verschiedenen Cluster in der allgemeinen Population mit Hauterkrankungen variieren.

Obwohl ein Zusammenhang zwischen Clustern und Akzeptanzraten festgestellt wurde, zum Beispiel eine hohe Akzeptanz bei den „Digitalen Enthusiasten” und eine geringe Akzeptanz bei den „Digitalen Skeptikern”, lässt sich dies nicht auf die Zukunft übertragen. Erstens verhindert das Querschnittsdesign, die Richtung des festgestellten Zusammenhangs zu bestimmen. Zweitens ist die Befragung anfällig für den *Intention–Behavior Gap*.^52^ Selbst wenn Akzeptanz die Anwendung fördert, muss die festgestellte Absicht, DHI in Zukunft zu nutzen, nicht unbedingt zu einem tatsächlichen Anstieg führen.

Für die Datenreduktion wurde eine explorative Faktorenanalyse anstelle einer konfirmatorischen Faktorenanalyse gewählt, da keine theoretischen Annahmen über Item‐Interaktionen getroffen wurden. Der explorative Ansatz schränkt die Vergleichbarkeit mit anderen Untersuchungen zur Akzeptanz ein, die häufig das UTAUT‐Framework als methodische Grundlage verwenden.^27^, ^53^ Eine weitere Einschränkung besteht darin, dass, wie empfohlen, keine konfirmatorische Faktorenanalyse durchgeführt wurde, da die Stichprobengröße eine Aufteilung für eine solche Analyse nicht zuließ.^54^ Trotz dieser Einschränkung wurden die EFA‐Berechnungen auf der Grundlage des KMO‐Kriteriums und des Bartlett‐Sphärizitätstests als angemessen erachtet, was zu Faktoren mit akzeptabler interner Konsistenz führte.^34^

## Schlussfolgerungen

Die Ergebnisse unserer Befragung zeigten, dass Patienten mit chronischen Hauterkrankungen vier verschiedenen Typen von digitalen Nutzern zugeordnet werden können. Die meisten Patienten innerhalb der identifizierten Cluster sind bereit, DHI in Zukunft häufiger zu nutzen. Wir stellten eine hohe Akzeptanz fest. Die derzeitige Nutzung von Interventionen ist jedoch gering bis mäßig, was wahrscheinlich auf mehrere Barrieren zurückzuführen ist, darunter die begrenzte Verfügbarkeit solcher DHI, die nicht ausreichend in das Gesundheitssystem integriert sind oder von diesem erstattet werden. Datenschutzbedenken und begrenzte digitale Kompetenzen der Patienten sollten von Entwicklern, Forschern und sogar Krankenkassen anerkannt werden, um die Akzeptanz von DHI zu verbessern und Unterschiede beim Zugang zu DHI zu verringern. Letzteres kann zu Ungleichheiten in der digitalen Gesundheit führen. Die eher skeptischen Patienten, verdienen in der Praxis besondere Aufmerksamkeit, einschließlich einer stärkeren Befähigung und gemeinsamer Entscheidungen vor der Nutzung digitaler Technologien. Darüber hinaus sollten die Akzeptanz und die Fähigkeiten von Ärzten im Umgang mit DHI verbessert werden, da auch ihre Meinung eine Barriere darstellen kann. Laufende Forschungsarbeiten und gemeinsame Anstrengungen sind unerlässlich, um die Akzeptanz und Inklusivität digitaler Gesundheitslösungen in der Dermatologie zu verbessern.

## DANKSAGUNG

Die Autoren danken dem Wissenschaftskommunikationsteam des IVDP, insbesondere Paula Willer, für die redaktionelle Bearbeitung. Wir danken dem Open‐Access‐Publikationsfonds des UKE – Universitätsklinikum Hamburg‐Eppendorf für die finanzielle Unterstützung.

Open access Veröffentlichung ermöglicht und organisiert durch Projekt DEAL.

## FINANZIERUNG

Dieses Forschungsprojekt wurde durch einen Forschungszuschuss der Novartis Pharma GmbH finanziell unterstützt. Novartis hatte keinen Einfluss auf die Konzeption, Datenerhebung, Analyse, Veröffentlichungsentscheidung oder Entwicklung dieser Studie.

## INTERESSENKONFLIKT

M.O. ist Mitautorin der deutschen AWMF‐Leitlinie zur Teledermatologie. M.A. ist Mitautor der deutschen AWMF‐Leitlinie zur Teledermatologie und wissenschaftlicher Berater für die Teledermatologie‐Plattformen derma2go AG, A+ Videoclinic GmbH und Novartis Pharma GmbH. P.R. erklärt, dass kein Interessenkonflikt besteht.

## Supporting information



Supporting Information

Supporting Information

Supporting Information

Supporting Information

Supporting 5Information
