# Supplementary material for: Einstellungen dermatologischer Patienten gegenüber digitalen Gesundheitsinterventionen: Eine Querschnittsbefragung und Clusteranalyse
Source: J Dtsch Dermatol Ges. 2026 Sep 8;24(9):1264–76. [Article in German] doi: 10.1111/ddg.70010x (PMC13551141; doi:10.1111/ddg.70010x)
Supplement: Supplementary file 2 [file DDG-24-1264-s001.docx]

# **Supplement 3 – Ergebnisse der explorativen Faktorenanalyse**

Tabelle S4 Faktorladungen der explorativen Faktorenanalyse

| Item | Text | Ladung | Missings  N (%) |
| --- | --- | --- | --- |
| Faktor 1: Akzeptanz von DHIs und Vertrauen (Alpha: 0,89) | | |  |
| 1 | Ich könnte mir vorstellen vermehrt digitale Anwendungen für meine Erkrankung zu nutzen. | 0,805 | 6 (1,7 %) |
| 3 | Ich würde digitale Anwendungen anderen Patienten mit einer ähnlichen Erkrankung empfehlen. | 0,746 | 7 (2,0 %) |
| 2 | Ich würde digitale Anwendungen regelmäßig nutzen, wenn sie mir empfohlen würden. | 0,709 | 4 (1,2 %) |
| 13 | Ich könnte mir vorstellen von mir digital gesammelte Daten mit meinem behandelnden Arzt zu besprechen. | 0,654 | 3 (0,9 %) |
| 14 | Meine gesammelten digitalen Daten müssten von mir individuell für meinen behandelnden Arzt freigegeben warden können. | 0,642 | 6 (1,7 %) |
| 18 | Digitale Anwendungen können unnötige Wege für mich als Patient einsparen. | 0,489 | 7 (2,0 %) |
| 11 | Personen in meinem direkten Umfeld würden es befürworten, wenn ich digitale Anwendungen nutzen würde. | 0,470 | 6 (1,7 %) |
| 19 | Digitale Anwendungen können eine sinnvolle Ergänzung zu meiner bestehenden Versorgung sein. | 0,443 | 6 (1,7 %) |
| 10 | Ich würde den Empfehlungen eine Patientenselbsthilfeverbandes in Bezug auf digitale Anwendungen vertrauen. | 0,428 | 6 (1,7 %) |
| 9 | Ich würde den Empfehlungen von anderen Patienten in Bezug auf digitale Anwendungen vertrauen. | 0,413 | 5 (1,5 %) |
| Faktor 2: Digitale Gesundheitskompetenzen der Patienten (Alpha: 0,82)* | | | |
| 26 | Ich habe exzellente Fähigkeiten im Umgang mit digitalen Medien (z. B. PC, Smartphone, Tablet). | -0,662 | 8 (2,3 %) |
| 27 | Ich fühle mich sicher bei der Nutzung digitaler/Online-Anwendungen. | -0,657 | 7 (2,0 %) |
| 5 | Mir würde es leichtfallen eine digitale Anwendung zu nutzen. | -0,569 | 5 (1,5 %) |
| 3 | Ich kann im Internet zuverlässige von fragwürdigen Informationen unterscheiden. | -0,507 | 6 (1,7 %) |
| 6 | Ich könnte digitale Anwendungen einfach in meinen Alltag integrieren. | -0,408 | 6 (1,7 %) |
| 30 | Ich weiß, wo ich im Internet nützliche Gesundheitsinformationen finden kann. | -0,404 | 5 (1,5 %) |
| Faktor 3: Unsicherheiten bezogen auf DHIs (Alpha: 0,79) | | | |
| 16 | Digitale Anwendungen können mich in Hinblick auf meine Erkrankung verunsichern. | 0,832 | 8 (2,3 %) |
| 17 | Digitale Anwendungen können die Erkrankung zu sehr zum Mittelpunkt meines Alltags machen. | 0,671 | 4 (1,2 %) |
| 1 | Ich mache mir Sorgen, dass erhobene Daten missbraucht werden. | 0,575 | 6 (1,7 %) |
| 28 | Das Internet ist etwas Bedrohliches für mich. | 0,532 | 11 (3,2 %) |
| 29 | Ich habe Angst, bei der Nutzung digitaler/Online-Anwendungen einen nicht korrigierbaren Fehler zu machen. | 0,466 | 9 (2,6 %) |
| Faktor 4: Positive Auswirkungen von DHIs (Alpha: 0,91) | | | |
| 21 | Digitale Anwendungen können mir helfen mich besser mit meiner Krankheit auseinanderzusetzen. | 0,901 | 8 (2,3 %) |
| 22 | Digitale Anwendungen können mir helfen, meine Eigenverantwortung bei der Behandlung zu stärken. | 0,900 | 9 (2,6 %) |
| 20 | Digitale Anwendungen können das Vertrauen in meine Therapie stärken. | 0,714 | 9 (2,6 %) |
| 23 | Digitale Anwendungen können mir helfen mich besser auf eine Sprechstunde mit meinem Arzt vorzubereiten. | 0,676 | 9 (2,6 %) |
| Aufgrund geringer Faktorladungen < 0,4 ausgeschlossene Variablen | | | |
| 4 | Ich wäre bereit, für digitale Anwendungen zu zahlen. | -0,305 | 4 (1,2 %) |
| 24 | Digitale Anwendungen können helfen die Arzt-Patienten-Kommunikation zu verbessern. | 0,337 | 10 (2,9 %) |
| 25 | Digitale Anwendungen vermindern den persönlichen Kontakt zu meinem behandelnden Arzt. | -0,204 | 10 (2,9 %) |
|  | Mein behandelnder Arzt würde den Einsatz digitaler Anwendungen befürworten. | 0,252 | 12 (3,5 %) |
| 8 | Ich würde den Empfehlungen meines behandelnden Arztes in Bezug auf digitale Anwendungen vertrauen. | 0,377 | 7 (2,0 %) |
| 7 | Ich finde es schwierig seriöse von unseriösen digitalen Anwendungen zu unterscheiden. | -0,383 | 10 (2,9 %) |
| Kaiser-Meyer-Olin-Maß für die Angemessenheit der Stichprobe: 0,90  Bartlett-Test auf Sphärizität: X² (df: 171) = 3335, p < 0,001  Rotationsmethode: Oblimin | | | |

*Die Polarität wurde für diesen Faktor beibehalten; daher weisen positive Ladungen weiterhin auf eine höhere digitale Kompetenz hin (z. B. hat der Punkt „Ich verfüge über ausgezeichnete Fähigkeiten im Umgang mit digitalen Medien” eine positive Ladung, sodass eine stärkere Zustimmung eine höhere digitale Kompetenz widerspiegelt).

Abbildung S1 -Screeplot der explorativen Faktorenanalyse

Tabelle S5 Gesamte erklärte Varianz und Eigenwerte der identifizierten Faktoren

| Faktor | Eigenwert | Erklärter Anteil der Varianz | Kumulativer Prozentsatz der erklärten Varianz |
| --- | --- | --- | --- |
| 1 | 7,409 | 38,995 | 38,995 |
| 2 | 2,196 | 11,556 | 50,551 |
| 3 | 1.273 | 6.699 | 57.250 |
| 4 | 1.142 | 6.011 | 63.261 |
| 5 | 0,981 | 5,161 | 68,423 |
| 6 | 0,935 | 4,919 | 73,342 |
| 7 | 0,676 | 3,560 | 76,902 |
| 8 | 0,633 | 3,331 | 80,233 |
| 9 | 0,545 | 2,867 | 83,100 |
| 10 | 0,504 | 2,655 | 85,755 |
| 11 | 0,463 | 2,436 | 88,192 |
| 12 | 0,428 | 2,255 | 90,447 |
| 13 | 0,365 | 1,923 | 92,369 |
| 14 | 0,314 | 1,653 | 94,022 |
| 15 | 0,291 | 1,529 | 95,551 |
| 16 | 0,289 | 1,521 | 97,073 |
| 17 | 0,204 | 1,071 | 98,144 |
| 18 | 0,181 | 0,954 | 99,098 |
| 19 | 0,171 | 0,902 | 100,000 |
| 20 | 0,265 | 1,062 | 96,316 |
| 21 | 0,244 | 0,977 | 97,293 |
| 22 | 0,207 | 0,827 | 98,120 |
| 23 | 0,178 | 0,710 | 98,830 |
| 24 | 0,166 | 0,664 | 99,494 |
| 25 | 0,126 | 0,506 | 100,000 |
