# Supplementary material for: Einstellungen dermatologischer Patienten gegenüber digitalen Gesundheitsinterventionen: Eine Querschnittsbefragung und Clusteranalyse
Source: J Dtsch Dermatol Ges. 2026 Sep 8;24(9):1264–76. [Article in German] doi: 10.1111/ddg.70010x (PMC13551141; doi:10.1111/ddg.70010x)
Supplement: Supplementary file 3 [file DDG-24-1264-s002.docx]

**Online Supplement S4**

Tabelle S6: Soziodemografische Daten der Stichprobe nach Indikation im Vergleich zur (dermatologischen) Population

|  | **Innerhalb unserer Studie** | | | **Prävalenz in der deutschen Bevölkerung** | | |
| --- | --- | --- | --- | --- | --- | --- |
| **Indikation**^1^ | % | % weiblich | Durchschnittsalter | % | % weiblich | Durchschnittsalter |
| **Psoriasis**^1^ | 39,0 | 59,2 | 60,2 | 2,5 | 43,4 | 43,2 |
| **Atopische Dermatitis** | 23,3 | 88,6 | 47,6 | 3,5 | 43,2 | 43,2 |
| **Vitiligo**^3^ | 9,0 | 58,1 | 52,0 | 0,52 | 43,5 | 43,1 |
| **Hautkrebs**^2^ | 7,6 | 88,5 | 55,0 | MM*: 0,22  NMSC**: 3,1 | 41,5 | 43,0 |
| **Hidradenitis**^1^ **suppurativa** | 21,2 | 86,3 | 43,8 | 1,0–2,0 | 47,7 | 43,6 |

*MM: Malignes Melanom

**NMSC: Nicht-melanozytärer Hautkrebs

^1^Hansen I, Augustin M, Schäfer I, Mohr N: Epidemiologie von Hautkrankheiten in Deutschland: systematische Literaturanalyse des aktuellen Forschungsstands – Teil 2: nichtinfektiöse Hauterkrankungen [Epidemiology of Skin Diseases in Germany: Systematic Review of the Current State of Research - Part 2: Non-infectious Skin Diseases]. J Dtsch Dermatol Ges 2022; 20 (4): 445-457.

^2^Hansen I, Augustin M, Schäfer I, Mohr N: Epidemiologie von Hautkrankheiten in Deutschland: systematische Literaturanalyse des aktuellen Forschungsstands - Teil 1: Tumorerkrankungen der Haut [Epidemiology of Skin Diseases in Germany: Systematic Review of the Current State of Research - Part 1: Cutaneous Tumor Diseases]. J Dtsch Dermatol Ges 2022; 20 (3): 257-271.

^3^Mohr N, Petersen J, Kirsten N, Augustin M: Epidemiology of Vitiligo – A Dual Population-Based Approach. Clinical Epidemiology 2021:13; 373-382.
