# Supplementary material for: Einstellungen dermatologischer Patienten gegenüber digitalen Gesundheitsinterventionen: Eine Querschnittsbefragung und Clusteranalyse
Source: J Dtsch Dermatol Ges. 2026 Sep 8;24(9):1264–76. [Article in German] doi: 10.1111/ddg.70010x (PMC13551141; doi:10.1111/ddg.70010x)
Supplement: Supplementary file 4 [file DDG-24-1264-s003.docx]

# **Supplement 5 – Gesamtergebnisse**

Tabelle S7 Zustimmung pro Item in der Gesamtgruppe (n = 344)

| Punkt | Text | Missings  N (%) | Stimme eher zu / Stimme völlig zu  N (%) |
| --- | --- | --- | --- |
| Faktor 1: Akzeptanz von DHIs und Vertrauen | | | |
| 1 | Ich könnte mir vorstellen vermehrt digitale Anwendungen für meine Erkrankung zu nutzen. | 6 (1,7 %) | 260 (76,9 %) |
| 3 | Ich würde digitale Anwendungen anderen Patienten mit einer ähnlichen Erkrankung empfehlen. | 7 (2,0 %) | 221 (65,6 %) |
| 2 | Ich würde digitale Anwendungen regelmäßig nutzen, wenn sie mir empfohlen würden. | 4 (1,2 %) | 260 (76,5 %) |
| 1 | Ich könnte mir vorstellen von mir digital gesammelte Daten mit meinem behandelnden Arzt zu besprechen. | 3 (0,9 %) | 252 (75,2 %) |
| 14 | Meine gesammelten digitalen Daten müssten von mir individuell für meinen behandelnden Arzt freigegeben werden können. | 6 (1,7 %) | 266 (79,2 %) |
| 18 | Digitale Anwendungen können unnötige Wege für mich als Patient einsparen. | 7 (2,0 %) | 264 (78,3 %) |
| 11 | Personen in meinem direkten Umfeld würden es befürworten, wenn ich digitale Anwendungen nutzen würde. | 6 (1,7 %) | 108 (32,0 %) |
| 19 | Digitale Anwendungen können eine sinnvolle Ergänzung zu meiner bestehenden Versorgung sein. | 6 (1,7 %) | 279 (82,5 %) |
| 10 | Ich würde den Empfehlungen eines Patientenselbsthilfeverbandes in Bezug auf digitale Anwendungen vertrauen. | 6 (1,7 %) | 273 (79,4 %) |
| 9 | Ich würde den Empfehlungen von anderen Patienten in Bezug auf digitale Anwendungen vertrauen. | 5 (1,5 %) | 158 (46,6 %) |
| Faktor 2: Digitale Gesundheitskompetenzen der Patienten | | | |
| 2 | Ich habe exzellente Fähigkeiten im Umgang mit digitalen Medien (z. B. PC, Smartphone, Tablet). | 8 (2,3 %) | 239 (71,1 %) |
| 27 | Ich fühle mich sicher bei der Nutzung digitaler/Online-Anwendungen. | 7 (2,0 %) | 277 (82,2 %) |
| 5 | Mir würde es sehr leichtfallen eine digitale Anwendung zu nutzen. | 5 (1,5 %) | 246 (72,6 %) |
| 3 | Ich kann im Internet zuverlässige von fragwürdigen Informationen unterscheiden. | 6 (1,7 %) | 251 (74,3 %) |
| 6 | Ich könnte digitale Anwendungen einfach in meinen Alltag integrieren. | 6 (1,7 %) | 251 (74,3 %) |
| 30 | Ich weiß, wo ich im Internet nützliche Gesundheitsinformationen finden kann. | 5 (1,5 %) | 294 (86,7 %) |
| Faktor 3: Unsicherheiten bezogen auf DHIs | | | |
| 16 | Digitale Anwendungen können mich in Hinblick auf meine Erkrankung verunsichern. | 8 (2,3 %) | 148 (43,9 %) |
| 17 | Digitale Anwendungen können die Erkrankung zu sehr zum Mittelpunkt meines Alltags machen. | 4 (1,2 %) | 81 (24,2 %) |
| 15 | Ich mache mir Sorgen, dass erhobene Daten missbraucht werden. | 6 (1,7 %) | 148 (43,9 %) |
| 28 | Das Internet ist etwas Bedrohliches für mich. | 11 (3,2 %) | 16 (4,8 %) |
| 29 | Ich habe Angst, bei der Nutzung digitaler/Online-Anwendungen einen nicht korrigierbaren Fehler zu machen. | 9 (2,6 %) | 30 (9,0 %) |
| Faktor 4: Positive Auswirkungen von DHIs | | | |
| 21 | Digitale Anwendungen können mir helfen mich mehr mit meiner Erkrankung auseinanderzusetzen. | 8 (2,3 %) | 176 (52,4 %) |
| 22 | Digitale Anwendungen können mir helfen, meine Eigenverantwortung bei der Behandlung zu stärken. | 9 (2,6 %) | 190 (56,7 %) |
| 20 | Digitale Anwendungen können das Vertrauen in meine Therapie stärken. | 9 (2,6 %) | 175 (52,4 %) |
| 2 | Digitale Anwendungen können mir helfen mich besser auf eine Sprechstunde mit meinem Arzt vorzubereiten. | 9 (2,6 %) | 232 (69,3 %) |
| Aufgrund geringer Faktorladungen von < 0,4 ausgeschlossene Variablen | | | |
| 4 | Ich wäre bereit, für digitale Anwendungen zu zahlen. | 4 (1,2 %) | 64 (18,8 %) |
| 2 | Digitale Anwendungen können helfen die Arzt-Patienten-Kommunikation zu verbessern. | 10 (2,9 %) | 180 (53,9 %) |
| 25 | Digitale Anwendungen vermindern den persönlichen Kontakt zu meinem behandelnden Arzt. | 10 (2,9 %) | 164 (49,1 %) |
| 12 | Mein behandelnder Arzt würde den Einsatz digitaler Anwendungen befürworten. | 12 (3,5 %) | 55 (16,6 %) |
| 8 | Ich würde den Empfehlungen meines behandelnden Arztes in Bezug auf digitale Anwendungen vertrauen. | 7 (2,0 %) | 268 (79,5 %) |
| 7 | Ich finde es schwierig seriöse von unseriösen digitalen Anwendungen zu unterscheiden. | 10 (2,9 %) | 103 (30,4 %) |

Tabelle S8 Nutzung von DHI in den letzten 12 Monaten (n = 344)

|  | Missings  N (%) | In den letzten 12 Monaten verwendet  N (%) |
| --- | --- | --- |
| Live Online-Videosprechstunde | 4 (1,2 %) | 7 (2,1 %) |
| Nachrichten mit dem behandelnden Arzt (Instant Messenger) | 5 (1,5 %) | 9 (2,6 %) |
| Online-Hautarztangebot (Store-and-Forward) | 4 (1,2 %) | 14 (4,1 %) |
| Elektronisches Patiententagebuch | 5 (1,5 %) | 18 (5,2 %) |
| Elektronische Patientenakte der Krankenkasse | 7 (2,0 %) | 45 (13,1 %) |
| Elektronische Medikamentenerinnerung | 8 (2,3 %) | 66 (19,2 %) |
| Online-Pollenflugvorhersage | 7 (2,0 %) | 108 (31,4 %) |
| Online-Erfahrungsaustausch mit Patienten | 4 (1,2 %) | 138 (40,1 %) |
| E-Mail-Kommunikation mit dem behandelnden Arzt | 4 (1,2 %) | 98 (28,5 %) |
| Elektronische Terminerinnerung | 5 (1,5 %) | 184 (53,5 %) |
| Online-Informations- und Aufklärungsportale | 7 (2,0 %) | 219 (63,7 %) |

Tabelle S9 Mögliche zukünftige Nutzung von DHIs (n = 344)

| Art der Intervention | Missings  N (%) | Stimme eher zu / Stimme völlig zu  N (%) |
| --- | --- | --- |
| Patienten, die sich vorstellen können, in Zukunft ein DHI zu nutzen: | | |
| Nachrichten mit dem behandelnden Arzt (Instant Messenger) | 7 (2,0 %) | 180 (52,3 %) |
| Online-Pollenflugvorhersage | 11 (3,2 %) | 189 (54,9 %) |
| Online-Erfahrungsaustausch mit Patienten | 5 (1,5 %) | 200 (58,1 %) |
| Online-Hautarztangebot (Store-and-Forward) | 5 (1,5 %) | 213 (61,9 %) |
| Elektronische Medikamentenerinnerung | 9 (2,6 %) | 217 (63,1 %) |
| Elektronisches Patiententagebuch | 9 (2,6 %) | 234 (68,0 %) |
| Live Online-Videosprechstunde | 5 (1,5 %) | 235 (68,3 %) |
| Elektronische Patientenakte der Krankenkasse | 10 (2,9 %) | 237 (68,9 %) |
| E-Mail-Kommunikation mit dem behandelnden Arzt | 6 (1,7 %) | 252 (73,3 %) |
| Elektronische Terminerinnerung | 9 (2,6 %) | 276 (80,2 %) |
| Online-Informations- und Bildungsportale | 9 (2,6 %) | 294 (85,5 %) |
